# A Long-Wavelength Fluorescent Squarylium Cyanine Dye Possessing Boronic Acid for Sensing Monosaccharides and Glycoproteins with High Enhancement in Aqueous Solution

**DOI:** 10.3390/s120505420

**Published:** 2012-04-27

**Authors:** Shingo Saito, Tara L. Massie, Takeshi Maeda, Hiroyuki Nakazumi, Christa L. Colyer

**Affiliations:** 1 Graduate School of Science and Engineering, Saitama University, 255 Shimo-Okubo, Sakura-ku, Saitama 338-8570, Japan; E-Mail: shingo@mail.saitama-u.ac.jp; 2 Department of Chemistry, Wake Forest University, Winston-Salem, NC 27109, USA; E-Mail: taralmassie@gmail.com; 3 Graduate School of Engineering, Osaka Prefecture University, 1-1 Gakuen-cho, Naka-ku, Sakai, Osaka 599-8531, Japan; E-Mails: tmaeda@chem.osakafu-u.ac.jp (T.M.); nakazumi@chem.osakafu-u.ac.jp (H.N.)

**Keywords:** squarylium dye, boronic acid, monosaccharide, glycoprotein, mucin, long wavelength fluorescence

## Abstract

Fluorescence sensing of saccharides and glycoproteins using a boronic acid functionalized squarylium cyanine dye (“**SQ-BA**”) is characterized in terms of synthetic, fluorometric, thermodynamic and kinetic parameters. In our previous work, this newly synthesized dye was successfully applied to the separation and quantification of Gram-positive bacteria by capillary electrophoresis with laser-induced fluorescence detection (CE-LIF); however, the fundamental properties of the dye and its saccharide complexes still required elucidation, as presented in this paper. The dye itself forms nonemissive, soluble aggregates in aqueous solution. With the addition of a monosaccharide, the dye aggregate dissociates to form an emissive monomer accompanied by the formation of a cyclic *cis*-diol ester with long-wavelength emission (*λ*_ex_ = 630 nm, *λ*_em_ = 660 nm). A very large fluorescence enhancement factor of 18× was observed for the sensing dye as a fructose complex at pH 10, yielding a limit of detection of 10 μM fructose. The relative order of fluorescence enhancement of SQ-BA with other monosaccharides was found to be: fructose > ribose > arabinose ≈ galactose > xylose > mannose > rhamnose > fucose ≈ glucose; and apparent affinity constants of 10^2.80^, 10^2.08^ and 10^0.86^ M^−1^ were determined for fructose, ribose and glucose, respectively. Formation of the emissive complexes occurred within minutes, proving the kinetics of the sugar-dye interactions to be suitable for on-column labeling methods in CE-LIF. Furthermore, the sensing dye was successfully applied to glycoproteins, mucin type I–S and type III, which were detected with high sensitivity in batch aqueous solution as a result of the sugar-selective boronic acid-diol esterification as well as hydrophobic interactions.

## 1. Introduction

A large number of synthetic fluorescent sensors for monosaccharides and sugar-containing biomaterials have been reported [1–3], many of which have involved interactions between boronic acid moeities and carbohydrates via the formation of cyclic *cis*-diol esters. Although this approach for carbohydrate recognition is effective, some challenges still remain. First, the recognition of saccharides in aqueous solution is desirable, while most reports of emissive probes employed aqueous-organic mixed solvents. Second, a high enhancement factor for carbohydrates is essential in order to detect low concentrations of saccharides (<10^−3^ M). Since boric acid- and boronic acid-based fluorescent probes generally have low binding constants with monosaccharides (in the range of 10 to 100 M^−1^), sensitive detection has proven difficult without a high emission enhancement factor. Third, the detection of sensor emission in the visible range may be hampered by unstable baselines and interferences from coexisting species in complex samples. While some recent reviews summarized a number of boronic acid-based fluorescent probes [1,4], many of the probes recognized carbohydrates via quenching mechanisms in aqueous-organic mixed solutions. In addition, to our knowledge there is no emissive probe documented to have a fluorescence enhancement factor of more than 2× upon complexation. Finally, with respect to detection wavelength, a symmetric squaraine (SQ) dye with two boronic acid moiteties, responding at the longest wavelength (*λ*_em_ = 645 nm) was described by Kukrer *et al.* [5]; but this offered only 25% emission enhancement for fructose in EtOH/aqueous mixed solution.

The use of the squaraine (SQ) structure is very attractive when designing chemical fluorescent sensors and probes because of its large molar extinction coefficient (∼10^5^ L·mol^−1^·cm^−1^), moderate quantum yield of emission (∼0.3), and long emission wavelength in the 600–800 nm range. Although the SQ structure itself is nonresponsive to chemical species, it can be designed for molecular recognition by modification to add a responsive functional group specific to the target molecule, such as metal ions, hydrogen ion, and proteins [6]. Modification of a SQ dye with boronic acid to create an artificial fluorescent receptor for saccharides has been reported only once, as described above [5], and so the present work aims to further develop the potential of this important class of sensors.

**Scheme 1.**
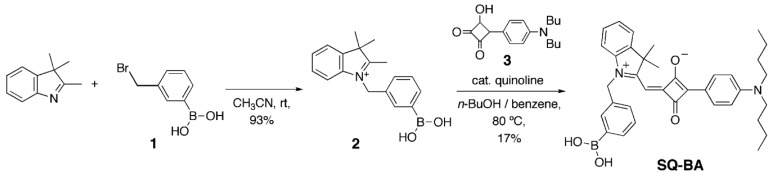
Synthesis of **SQ-BA**.

In our previous work, we reported a new capillary electrophoresis-laser-induced fluorescence detection (CE-LIF) methodology employing a boronic acid-modified SQ dye (abbreviated as **SQ-BA** in Scheme 1) as an on-column, fluorescent labeling agent for Gram-positive bacteria [7]. The SQ-BA dye successfully provided high fluorescence emission enhancement in the presence of fructose and Gram-positive bacteria in batch aqueous solutions at a long emission wavelength (*λ*_ex_ = 630 nm, *λ*_em_ = 660 nm). The sensitivity of this new dye towards Gram-positive bacteria was high enough to enable the detection of only three cells by our modified CE-LIF method. This high enhancement seemed to originate from boronate-*cis*-diol esterification with fructose or moieties present in the peptidoglycan layer in bacteria. While the feasibility of applying this dye to CE-LIF methods was demonstrated, basic properties of the dye and its saccharide or glycoprotein complexes were not investigated in terms of synthetic, thermodynamic, kinetic and fluorometric parameters, which are essential for sensing saccharides. Hence, our goal in this paper is to provide a detailed account of the chemical nature of the high-sensitivity, sugar-sensing dye, SQ-BA, which possesses a long emission wavelength (over 645 nm), a high enhancement factor (over 18×), and a high affinity towards monosaccharides and glycoproteins.

## 2. Experimental Section

### 2.1. Reagents

All starting materials for the synthesis of SQ-BA were purchased from Aldrich (Tokyo, Japan), Wako Pure Chemical Industries (Osaka, Japan), and Tokyo Kasei Kogyo (TCI, Tokyo, Japan). Silica gel (SiO_2_) for the flash chromatography was purchased from Kanto Chemical (Tokyo, Japan). 3-Bromomethylphenylboronic acid (**1**) and 3-(4-*N,N*-dibutylaminophenyl)-4-hydroxycyclobut-3-ene-1,2-dione (**3**) were synthesized according to the literature [8,9]. For spectrophotometric experiments, analytical grade reagents were employed exclusively. A carbonate buffer was prepared by dissolving an appropriate amount of sodium hydrogen carbonate purchased from Fisher Scientific (Fair Lawn, NJ, USA) in ultrapure water (>18 MΩ) by Milli-Q reagent water system (Millipore, Bedford, MA, USA) to a concentration of 0.1 M, prior to adjusting the buffer pH to 10.0 by addition of small portions of a concentrated sodium hydroxide solution. All carbohydrates (l-arabinose (ara), d-fructose (fru), l-fucose (fuc), d-galactose (gal), d-glucose (glc), d-mannose (man), l-rhamnose (rha), d-ribose (rib), d-xylose (xyl)) and glycoproteins (mucin type I–S from bovine submaxillary glands (bound sialic acid 9–17%) and mucin type III (bound sialic acid 0.5–1.5%, partially purified powder)) were purchased form Sigma-Aldrich (St. Louis, MO, USA). Stock solutions of 0.1–1.0 M monosaccharides and 1 mg/mL mucin proteins were prepared by dissolving the dry reagents in ultrapure water to the desired concentrations. These stock solutions were stored in a refrigerator when not in use. An appropriate amount of SQ-BA dye was dissolved in DMF to yield a 0.2 mM stock solution. A 10-fold dilution of this with ultrapure water yielded a 20 μM working stock solution of SQ-BA dye, which was stored at 4 °C to avoid decomposition. The DMF stock solution of SQ-BA dye was stable for more than 3 months in a refrigerator. The typical DMF concentration in final separation buffers containing 1 μM SQ-BA was 1.0 % v/v.

### 2.2. Apparatus

The NMR spectra were obtained using a JEOL ECX-400 spectrometer operating at 400 MHz for ^1^H. Chemical shifts are reported in parts per million (*δ*) downfield from tetramethylsilane (TMS) as the internal standard in DMSO-*d_6_*. The matrix-assisted laser desorption/ionization (MALDI) mass spectra were recorded using a Shimadzu AXIMA spectrometer (Kyoto, Japan), employing α-cyano-4-hydroxycinnamic acid (CHCA) as a matrix. The IR spectra were recorded using a Shimadzu Fourier Transform IR-8400S spectrophotometer. Absorbance spectra were recorded by an Agilent 8453 UV-visible spectroscopy system with a 1 cm cuvette. A Perkin-Elmer LB50B luminescence spectrophotometer was employed for emission and excitation spectra. The band pass and the photomultiplier voltage were set at 10 nm and 5 nm for excitation and emission, and 900 V, respectively. Samples were mixed and analyzed exclusively in quartz cuvettes (Perkin-Elmer) to avoid adsorption of the dye onto the vessel wall (vide infra).

### 2.3. Synthesis of the New Boronic Acid Functionalized SQ Dye (SQ-BA)

#### 2.3.1. Synthesis of 1-(3-Boronobenzyl)-2,3,3-Trimethyl-3H-Indolium Bromide (**2**)

3-Bromomethylphenylboronic acid (**1**, 0.50 gm 2.4 mmol) and 2,3,3-trimethyl-3*H*-indole (1.93 g, 13 mmol) were dissolved in acetonitrile (10 mL) and stirred at room temperature for 18 h. After removal of solvent, the residue was washed with hexane and dried *in vacuo* to give **2** as a red solid (0.99 g, 93%), which was used in the next step without further purification. ^1^H-NMR (DMSO-*d_6_*, 300 MHz): *δ* 7.86 (d, *J* = 6.4 Hz, 1H), 7.77 (d, *J* = 6.4 Hz, 1H), 7.70 (s, 1H), 7.63–7.54 (m, 1H), 7.50–7.25 (m, 4H), 5.81 (s, 2H), 2.96 (s, 3H), 1.60 (s, 6H).

#### 2.3.2. Synthesis of Boronic Acid Functionalized Squarylium Dye, **SQ-BA**

In a 200 mL, two-neck, round-bottom flask with condenser, **2** (0.20 gm 0.54 mmol) and 3-(4-dibutylaminophenyl)-4-hydroxycyclobut-3-ene-1,2-dione (**3**, 0.15 g, 0.49 mmol) were dissolved in a mixture of *n*-butanol (10 mL) and benzene (3 mL). Then a catalytic amount of quinoline (0.01 mL) was added and the solution was heated at 80 °C for 0.5 h. After cooling, the solvent was removed on a rotary evaporator, the residue was purified by silica gel column chromatography (eluent: CHCl_3_/MeOH, 10/1, v/v), and the product was precipitated from CHCl_3_/hexane solution to obtain **SQ-BA** as a green solid (51 mg, 17%). ^1^H-NMR (DMSO-*d_6_*, 300 MHz): *δ* 8.07 (s, 2H), 8.00 (d, *J* = 8.8 Hz, 2H), 7.71–7.62 (m, 3H), 7.45–7.31 (m, 5H), 6.82–6.79 (d, *J* = 8.8 Hz, 2H), 6.03 (s, 1H), 5.56 (s, 2H), 3.48–3.40 (m, 4H), 1.80 (s, 6H), 1.59–1.48 (m, 4H), 1.40–1.29 (m, 4H), 0.92 (t, *J* = 7.1 Hz, 6H). IR (KCl, cm^–1^): 3448, 1593, 1558, 1417, 1180. MS (MALDI): *m/z* calcd. for [M(C_36_H_41_BN_2_O_4_)]^+^, 576.32; found 576.51.

## 3. Results and Discussion

### 3.1. UV-VIS Absorption Spectra for the Free Dye and Its Complex with Fructose

**Figure 1.**
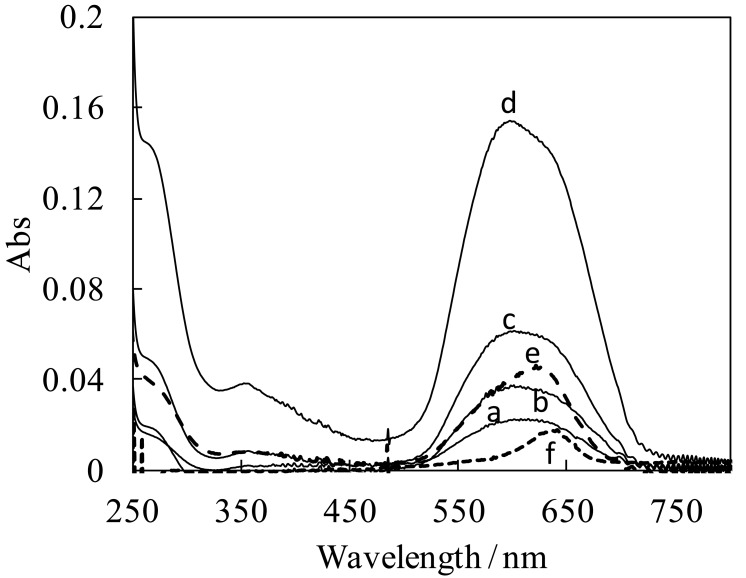
Absorption spectra for the free SQ-BA dye and its complex with fructose. Concentration of SQ-BA: (**a**) 6 × 10^−7^ M; (**b**) 1 × 10^−6^ M; (**c**) 2 × 10^−6^ M; (**d**) 4 × 10^−6^ M; (**e**) 1 × 10^−6^ M + 1 mM fructose; (**f**) 1 × 10^−6^ M in DMF. [carbonate] = 10 mM (pH 10.0).

The new squarylium dye bearing a boronic acid functional group (SQ-BA) was prepared by the condensation of indolenium with a 3-boronobenzyl group (2) and semisquarylium (3) using *n*-BuOH-benzene as solvent (according to Scheme 1). UV-VIS absorption spectra were measured for various concentrations of the resulting SQ-BA dye in the range of 1.0 × 10^−8^–4.0 × 10^−6^ M. As we previously reported [7], a broad band at around 550–700 nm was observed, as shown in Figure 1(a–d). This strongly suggests that the dye forms water-soluble aggregates, in agreement with results reported by other researchers [10,11], in which broadened bands of squarylium dye aggregates were observed, while a sharper band of monomer was observed accompanied with dissociation of the aggregates in organic solvents. In our case, a sharp band with maximum absorption at 637 nm (most likely attributable to soluble monomer) was observed in DMF solvent (Figure 1(f)).

With the addition of high concentration of fructose (1.0 × 10^−3^ M) to the 1 μM dye solution, the shape of the absorption spectrum clearly changed. The absorption band became sharper, and a red-shift of the absorption maximum from 595 nm to 628 nm was also observed (Figure 1(e)). These spectral changes indicate the formation of a cyclic *cis*-diol ester complex between the dye and fructose, and also imply its disaggregation from a soluble aggregate, as judged by the similarity of the absorption maxima at 628 nm relative to that in DMF (637 nm), where the dye exists as a monomer.

### 3.2. Fluorescent Properties of Free SQ-BA

**Figure 2.**
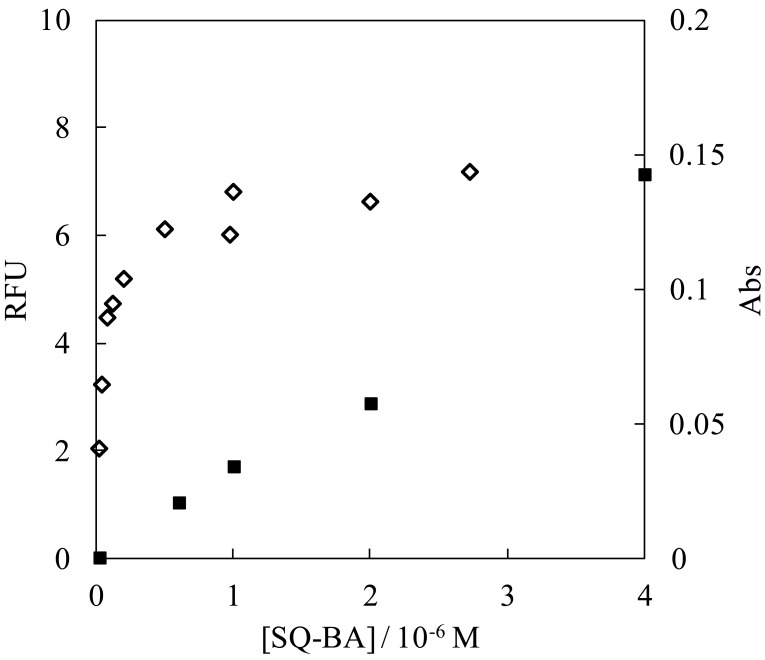
Effect of the dye concentration on fluorescence intensity (◊) and absorbance (■) in aqueous carbonate (10 mM, pH 10.0) buffer solution. *λ*_em_ = 660 nm, *λ*_abs_ = 630 nm. [SQ-BA] = 1.0 × 10^−8^–4 × 10^−6^ M.

The dye in aqueous solution showed very low fluorescence intensity (*λ*_ex_ = 630 nm, *λ*_em_ = 660 nm, Figure 2 and Figure 6(a)). This is possibly due to the formation of aggregates, as supported by UV-VIS spectra (vide supra), consistent with quenching processes reported for other SQ dyes [10,11]. Although such low emission from a blank dye sample is preferable to obtain a high signal-to-noise ratio for sensitive detection, the emission intensity for SQ-BA was irreproducible, and decreased over time when the sample solution of the dye was stored in glass or polypropylene wares. This could cause a serious problem for fluorescence detection since such fluctuation in the blank signal could lead to unstable measurements. The leading cause of this seemed to be adsorption of the dye onto the glass or polypropylene vessel wall: a pale blue stain could be seen even by the naked eye on the vessel walls after a long period of standing in contact with the dye solution. Fortunately, a stable fluorescence emission signal for the blank was obtained by using quartz wares, which indicated no adsorption. Although the exact mechanism of the adsorption remains unclear, the ability to achieve stable measurements by means of exclusive use of quartz wares negated further problems. A stable blank signal in a quartz cuvette can be seen in a time course study of the dye alone (*vida infra*, Figure 5(a)). Henceforth, quartz wares were exclusively used for all experiments.

The dependence of fluorescence intensity on concentration of the dye is depicted in Figure 2. The emission intensity was saturated for a high concentration of the dye ([SQ-BA] > 0.5 × 10^−6^ M), while the absorbance remained proportional to the dye concentration over the range studied (RFU = (3.5 ± 0.2) × 10^4^ [SQ-BA] − (0.002 ± 0.002), *R*^2^ = 0.99). No inner filter effect was confirmed, even at the highest concentration, under our experimental conditions, which provided absorbance <0.15. Equilibria of aggregated states of the dye seem to occur judging from the slight changes observed in the shape of the UV/VIS spectra with increasing dye concentration (Figure 1(a–d)). In particular, the blue shift of the maximum absorption wavelength with increasing dye concentration strongly indicates the existence of multiple dye species in solution. Complex, broadened spectra were reported for multiple aggregates of squarylium dyes by Fukuda and Nakahara [12]. These aggregation reactions possibly provide the saturation of the emission we observed. In terms of developing a sensing system, the saturation phenomenon is useful because the background signal remains suppressed at low levels even at high concentrations of the dye. In fact, 1 μM of the SQ-BA dye was employed for the determination of gram-positive bacteria in CE-LIF studies [7], which was high enough to ensure sufficient labeling of the analyte without jeopardizing the very stable baseline observed.

### 3.3. Emission Enhancement with the Addition of Monosaccharides

The fluorescence spectra of the dye in the presence of fructose were measured (*vide infra*, Figure 6(e)). While the dye aggregate showed weak emission (Figure 6(a)), the dye-fructose complex provided strong emission with wavelengths at 630 and 660 nm for excitation and emission, respectively. The excitation wavelength corresponds well to the absorption maxima at 628 nm seen in the UV/VIS spectrum (Figure 1(e)), which is also close to the absorption band of the dye monomer in DMF (637 nm in Figure 1(f)). These facts strongly support that the fructose-dye complex exists with the dye in its monomeric form. An enhancement factor of 18× was obtained at pH 10 in the presence of 20 mM fructose. It should be emphasized that, to our knowledge, the detection wavelength for the SQ-BA complex with fructose is the longest and the enhancement factor the largest compared to other fluorescent sensors for monosaccharides reported thus far.

**Figure 3.**
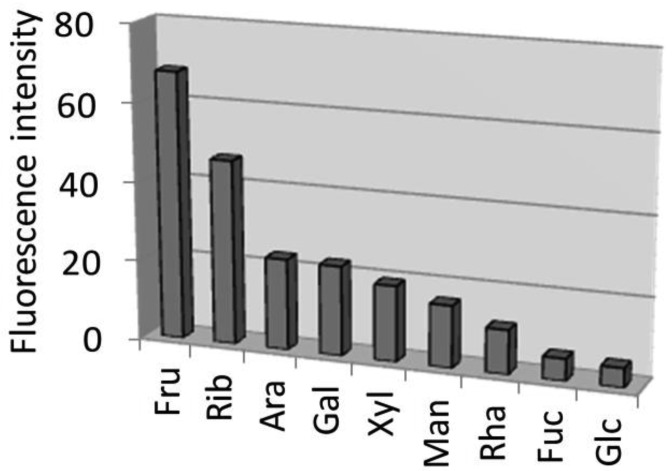
Fluorescence enhancement of SQ-BA with various monosaccharides. [SQ-BA] = 1 μM, [monosaccharide] = 5 mM, [carbonate] = 10 mM (pH 9.94).

Based on our previous work [7], the highest enhancement for the fructose—SQ-BA complex was observed at pH 10–11, and so the emission enhancement for other monosaccharides was also investigated at pH 10 (Figure 3). The shapes of excitation and emission spectra for other monosaccharides (data not shown) are the same as shown for the fructose-SQ-BA complex (Figure 6(e)). The relative order of fluorescence emission enhancement for the nine monosaccharides studied was: fru > rib > ara ≈ gal > xyl > man > rha > fuc ≈ glc. The highest selectivity towards fructose has been frequently reported for boronic acid-based fluorescent sensors due to their high thermodynamic stability [1,2].

### 3.4. Affinity Constants for Monosaccharide—SQ-BA Complexes

**Figure 4.**
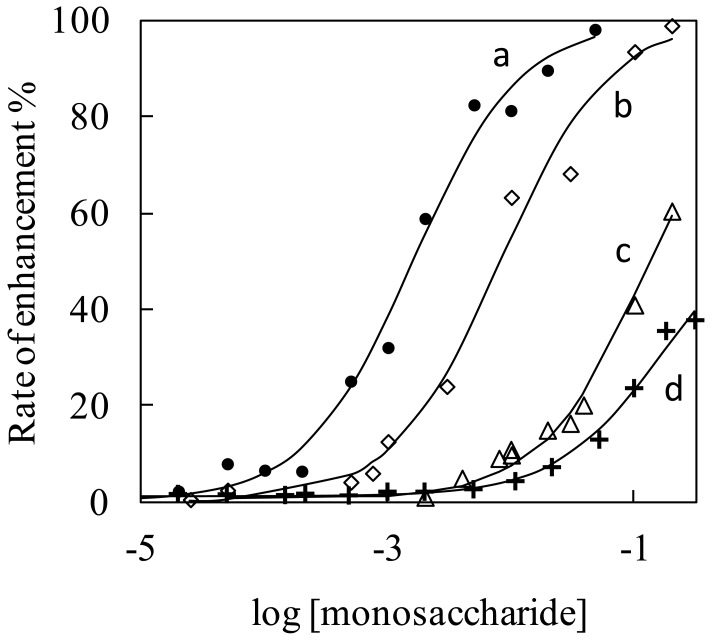
Titration experiments to determine the extent of monosaccharide−SQ-BA complex formation for: (**a**) fructose; (**b**) ribose; (**c**) glucose at pH 10.0; and (**d**) fructose at pH 7.4. The solid lines correspond to calculated non-linear, least-squares curve fits.

The affinity constants for SQ-BA complexes with monosaccharides were determined by a titration method, and the results are shown in Figure 4. The dependence of fluorescence intensity on monosaccharide concentrations resulted in sigmoidal curves. Non-linear, least-squares curve fitting of the plots agrees well with a one-to-one complex formation reaction process (solid lines in Figure 4). The affinity constants thus determined are summarized in Table 1. The order of the affinity constants (*K*_fru_ > *K*_rib_ > *K*_glc_), agrees with the order of the fluorescence enhancement observed for these three monosaccharides (see Figure 3). In addition, the affinity constant of the fructose—SQ-BA complex at pH 7.4 was significantly lower than at pH 10 (Table 1). These facts suggest that selectivity of the dye originates not from the quantum yield of the complex but from a particular affinity for the monosaccharide. The measured equilibrium constant for this new probe, SQ-BA, with fructose (631 M^−1^) is larger than equilibrium constants reported previously for conventional boronic acid receptors, which are on the order of 10 to 100 M^−1^ [1]. It should be noted that these constants are apparent values, since the formation equilibrium seems to be part of a complicated multi-equilibrium system along with aggregation reactions, as described earlier.

### 3.5. Kinetics for Complexation between SQ-BA Dye and Monosaccharides

**Figure 5.**
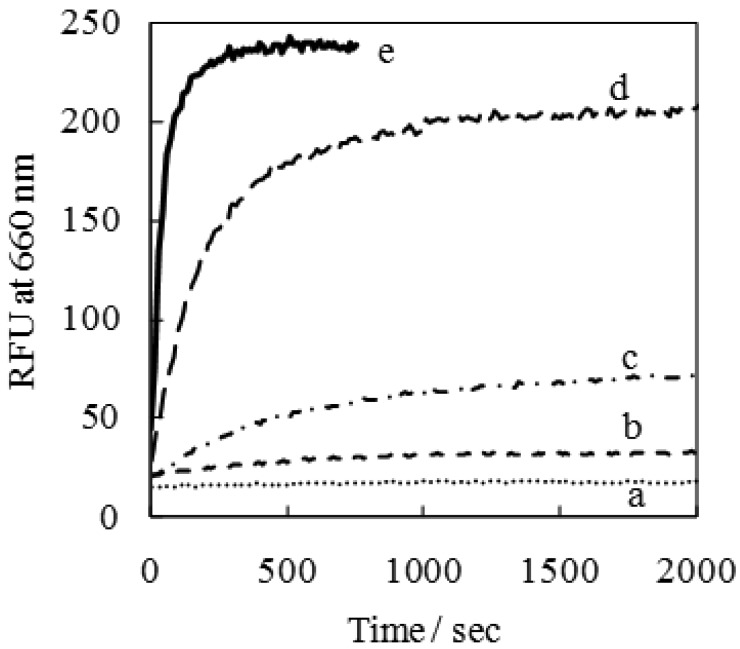
Time courses of emissive complexes of the dye ([SQ-BA] = 1 μM), with excess fructose: (**a**) [fructose] = 0, (**b**) 50 μM, (**c**) 0.5 mM, (**d**) 5 mM, and (**e**) 50 mM, prepared in carbonate buffer (10 mM, pH 9.94).

The time course for the formation reaction of the emissive fructose−dye complex was measured by mixing of 1 μM dye with a large excess of fructose (50 μM–50 mM), as shown in Figure 5. The time courses provided evidence of a first-order reaction with respect to the monosaccharide at the highest concentrations of fructose (Figure 5(d,e)), based on non-linear, least- squares curve fitting with a high correlation coefficient of R^2^ > 0.99 (data not shown).

This kinetic study reveals that the reaction between SQ-BA and sugar (at the highest concentrations of sugar) is largely completed within about 6 min, and in our previously published CE-LIF study [7], the target analyte (Gram-positive bacteria) was detected after 5–7 min. Hence, the present study confirms the suitability of this SQ-BA dye as an on-column labeling agent, which has important implications for additional method development for sugar-based analytes.

It is noteworthy that even the lowest concentration of fructose (50 μM) provides a detectable enhancement in SQ-BA fluorescence, as shown in Figure 5(b), and replicate experiments of emission spectra (data not shown). The detection limit for fructose (based on three times the standard deviation of the emission of the free dye) was calculated as 10μM.

### 3.5. Application to Mucin as a Model Glycoprotein

We designed this SQ-BA dye for glycoprotein sensing as well as for monosaccharide sensing. In our previous work, it was revealed that not only hydrophobic interactions, but also electrostatic and hydrogen bonding interactions have a significant effect on the noncovalent binding affinity between SQ dyes and proteins [13]. In particular, the binding properties of some carboxylate-modified SQ dyes with proteins were investigated previously, which strongly indicated that anionic carboxylate had a significant influence on the binding constants. Thus, it might be expected that this SQ-BA dye could cooperatively bind with glycoproteins not only through a targeted boronate-*cis*-diol interaction, but also through hydrophobic and hydrogen bonding interactions.

In the present work, two types of mucin, type I-S from bovine submaxillary glands and type III from porcine stomach, were examined as models of glycoproteins. Mucin is a glycoprotein with a large molecular weight (0.5–20 MDa) and large amounts of *O*-linked sugar chains (about 70–80% of mass) [14,15]. Mucin plays important roles in the defense against bacterial and enzymatic attack, and binding pathogens. Since mucin 1 (MUC 1) is known as a potentially useful cancer marker, analytical methods for its determination have been investigated by other researchers [16,17]. The *O*-linked carbohydrate chains in mucin are composed of a number of *N*-acetylglucosamine, *N*-acetylgalactosamine, fucose, galactose and sialic acid (*N*-acetylneuraminic acid) moieties, along with trace mannose. Unfortunately, the carbohydrate chains in mucin include little or no fructose, which we have shown to be most reactive with the SQ-BA dye.

**Figure 6.**
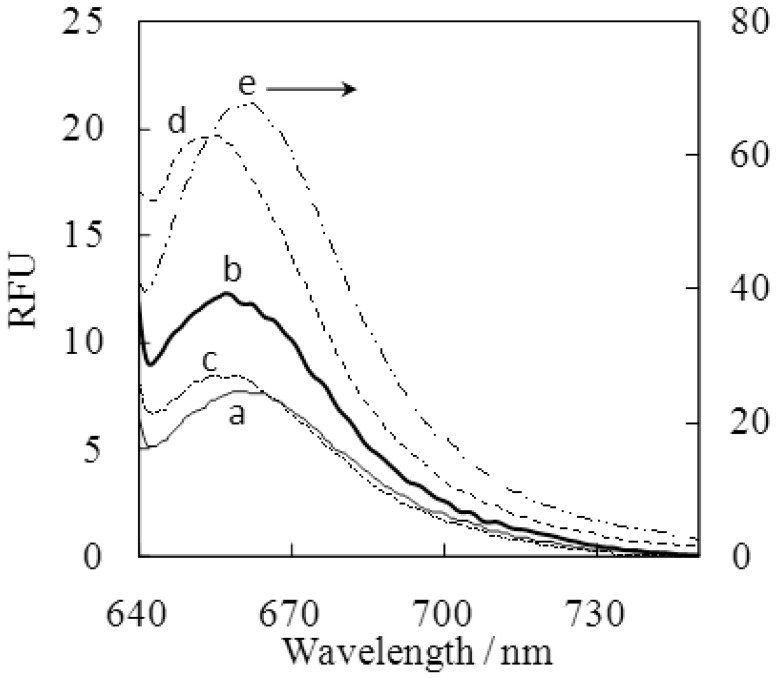
Emission spectra of SQ-BA (1.0 μM) complexes with mucin type I–S and type III proteins in carbonate buffer (10 mM, pH 10.0): (**a**) blank; (**b**) [mucin type I–S] = 50 μg/mL; (**c**) (b) + [borate] = 20 mM; (**d**) [mucin type III] = 50 μg/mL; (**e**) [fructose] = 10 mM.

When adding SQ-BA dye to sample solutions containing mucin proteins, a distinct enhancement of fluorescence was observed for type I–S and III (Figure 6(b,d)). This indicates that the dye could bind with the glycoproteins, despite the lack of fructose residues in their sugar chains. Slight differences in the maximum wavelength of emission were observed for monosaccharides, mucin type I–S, and type III complexes with SQ-BA: *λ*_em_ = 660–662 nm, 658 nm, and 655 nm for monosaccharides, type I–S, and type III, respectively. When a high concentration of borate (20 mM) was added to the protein/dye mixture, this served as a competing substance for sugar-chain binding sites, since the added borate could also form a cyclic ester via *cis*-diol interaction, and the resulting fluorescence intensity dramatically decreased (Figure 6(c)). This is a strong indication that binding of the SQ-BA dye with mucin could be substituted by binding with borate, and so we conclude that the SQ-BA dye must, at least in part, bind with mucin through saccharide-boronic acid interactions. Even though a large excess of borate was added, a slight fluorescence enhancement was still observed for the glycoprotein—SQ-BA sample mixture at a blue-shifted wavelength (654 nm, Figure 6(c)) relative to the emission of mucin complexes in the absence of added borate (Figure 6(d)). This fact suggests that the remaining enhancement originated not from a *cis*-diol type interaction, but from other interactions such as hydrophobic or hydrogen bonding. Since it was reported that mucin has hydrophobic protein core domains (∼20% of mass) [15], the SQ-BA dye could presumably bind to such domains, and so more than one kind of interactions would likely be involved in the binding mode between glycoproteins and SQ-BA dye. Even 5 μg/mL of mucin was successfully detected by the measured fluorescence enhancement. This concentration of mucin corresponds to 2.5 × 10^−10^–1.0 × 10^−8^ M.

## 4. Conclusions

The new dye, SQ-BA, has been shown to possess useful characteristics for sensing monosaccharides and glycoproteins. Both the dye's high enhancement factor (>18×) and its long emission wavelength (660 nm) are amongst the best emission properties reported for artificial fluorescent probes for saccharides thus far. Furthermore, there has been no previous demonstration of controlling such emission by on/off switching such as that reported herein, which arises from the addition of saccharides to induce the dissociation of soluble, nonemissive aggregates of the dye. Since this SQ-BA dye is not selective for a specific sugar, a separation system or sample pretreatment procedure will be necessary for detecting specific saccharides or sugar chains. It is noted that the above-mentioned properties are very suitable for application of the dye to separation techniques like CE-LIF. In the near future, we will design new SQ dye-based sensor molecules with boronic acid functional groups intended for specific analyte detection by CE-LIF.

## Figures and Tables

**Table 1.** Apparent affinity constants for 1:1 complexes between SQ-BA dye and monosaccharides.

	
	**pH 10** *^a^**^,^**^c^*	**pH 7.4** *^b^**^,^**^c^*
log *K*_fructose_	2.80 ± 0.08 *^d^*	0.76 ± 0.07
log *K*_ribose_	2.08 ± 0.11 *^d^*	—
log *K*_glucose_	0.86 ± 0.05	—

a *R*^2^ > 0.99,

b *R*^2^ > 0.97,

c errors were obtained from curve fitting calculation,

d from [7].
